# Multidimensional Poverty in Indonesia: Trend Over the Last Decade (2003–2013)

**DOI:** 10.1007/s11205-015-1044-0

**Published:** 2015-08-01

**Authors:** Wulung Hanandita, Gindo Tampubolon

**Affiliations:** Cathie Marsh Institute for Social Research, University of Manchester, Humanities Bridgeford Street Building 2F, Oxford Road, Manchester, M13 9PL UK

**Keywords:** Poverty assessment, Multidimensional poverty index, Indonesia, Susenas, Alkire–Foster method

## Abstract

The notion of poverty as an experience of multiple deprivation has been widely acknowledged. In Indonesia, however, poverty assessment has almost exclusively been conducted within the monetary space; even when multidimensionality is admitted, it has always been computed using variants of marginal method that are indifferent to joint deprivation. Applying a novel measurement method that is sensitive to both the incidence and the intensity of multiple deprivation to data from the National Socio-economic Survey (Susenas), this paper investigates the extent and the patterns of multidimensional poverty in Indonesia from 2003 to 2013 ($N=7{,}148{,}964$). An Indonesian version of the multidimensional poverty index is constructed by augmenting the existing consumption poverty measure with information on health and education. Results suggest that there was an unambiguous poverty reduction over the last decade at both national and sub-national levels. The data also reveal that progress has been inclusive across population subgroups, although spatial variation remains notable. The new poverty measurement method proves to be easily adaptable to the Indonesian context and could complement the methods currently employed by the Indonesian Statistical Bureau.

## Introduction

Income, or consumption poverty measures such as the World Bank’s dollar-a-day headcount ratio (Ravallion et al. 2009), is still the most prevalent measure of poverty used across the globe. However, from Asia to Africa (Batana 2013; Klasen 2000; Santos 2013; Ranis and Stewart 2012; Yu 2013), and across Europe to Latin America (Battison et al. 2013; Brandolini and D’Alessio 1998; Whelan et al. 2004), scholars have consistently documented that the lack of money is not always an accurate proxy for deprivations that society cares about. It has been argued that money metrics do not tell the whole story of human suffering, because poverty is not only about one’s inability to spend on essential goods and services. More than that, it is about one’s inability to enjoy valuable beings and doings (Sen 1985). Indeed, what is now generally accepted is a notion of poverty (or well-being for that matter) as an intrinsically multidimensional construct that encompasses the whole range of ways in which an individual can participate effectively in society.

Since the seminal works of Townsend (1979) and Sen (1985), different multidimensional poverty measures have been developed. Yet, as noted by Santos and Ura (2008: 1), ‘some of the proposed measures seem to have incorporated a multidimensional perspective at the cost of giving up the simplicity and intuition that characterise the unidimensional measures’. Statistical approaches to multidimensional poverty measurement (Filmer and Pritchett 2001; Sahn and Stifel 2003), for instance, rely on multivariate or latent-variable techniques to the extent that parameters are completely data-driven, leaving evaluators with limited control over the measure. Some axiomatic alternatives such as Bourguignon and Chakravarty (2003), on the other hand, satisfy a number of useful measurement properties but do strictly necessitate the availability of cardinal data; in reality, vital social indicators such as literacy and completion of primary school are usually ordinal in nature.

In an attempt to address these problems, Alkire and Foster (2011a; henceforth AF) proposed a new sort of multidimensional poverty measure: one that is not only simple to construct, but also retains many of the properties of the well-known Foster–Greer–Thorbecke (FGT) measures (Foster et al. 1984) of unidimensional poverty measurement. The AF method combines the FGT with the counting approach (Atkinson 2003), which is easy to understand and has a long history in sociology. The method deals with ordinal data in a straightforward manner by dichotomising individuals’ achievement into deprived and non-deprived states. Aggregation is then performed, first across deprivations experienced by each individual, and then across individuals, yielding a measure that is intuitively interpretable as the share of deprivations that poor individuals experience out of the total deprivations that the society could possibly experience.

As a generalisation of the classical FGT, the AF family of multidimensional poverty measures satisfies an array of desirable axioms (Alkire and Foster 2011a). Foremost among them are their ‘subgroup decomposition’ and ‘dimensional breakdown’ properties, which allow the overall poverty measure to be broken down into its social, geographical or dimensional constituents in a way that is both conceptually and technically defensible. A thorough characterisation of joint deprivations is further made possible by the availability of partial indices that capture the incidence as well as the intensity of poverty. The methodology is also transparent in the sense that all parameters are under the control of the evaluator, allowing normative decisions with regard to the selection of indicators, dimensional and poverty cut-offs, and weighting schemes to be easily incorporated into the analysis. In fact, acknowledging these novelties, in 2010 the United Nations Development Programme (UNDP) replaced its Human Poverty Index (HPI; first published in 1997) with the new multidimensional poverty index (MPI), based on the AF family of multidimensional measure (UNDP 2014, 2010: 95).

Applying the AF method to the National Socio-economic Survey data from Indonesia, this paper seeks to estimate the extent and to investigate the regional as well as the temporal patterns of multidimensional poverty in Indonesia for 11 consecutive years spanning from 2003 to 2013. The aim of this study is *not to replace* the official consumption poverty estimate with a new one, but rather *to augment* the conventional poverty measure with additional information on health and education using the same data source that has historically been used to estimate the official consumption poverty figure in Indonesia. This version of an Indonesian multidimensional poverty index (MPI) is constructed in a way that income poor individuals are ‘automatically’ multidimensionally poor, but not the converse. The present study considers the following questions: taking into account income, health and education dimensions, how many Indonesians are poor overall? Are urban areas always better off? Which island of the archipelago is the most deprived? Did recent progress, if any, benefit the poorest of the poor? And what happened to gender and spatial inequities during the last decade?

Indonesia, the world’s largest archipelagic state and the third-most populous developing country, is known for its exemplary achievement in terms of income poverty reduction and overall human development (Ranis and Stewart 2012). However, there is little research attempting to understand the nature of *simultaneous deprivations* experienced by its people. The majority of recent poverty evaluations have been conducted exclusively within the monetary space (Ilmma and Wai-Poi 2014; Strauss et al. 2004; Sumarto et al. 2014); even when multidimensionality is sought, it has always been computed using variants of marginal method (BPS 2015b; BPS et al. 2004) that are blind to joint deprivation (Alkire 2011: 503–504).

To date, only two studies attempted to measure the extent of simultaneous deprivations in Indonesia. Alkire and Foster (2011a), in the earliest showcase of their methodology, provided a national poverty estimate for the year 2007 using the Indonesia Family Life Survey data (IFLS; Thomas et al. 2012). But it is known that the IFLS sampling frame is not entirely representative of the population (RAND 2007); it neglects individuals living in the eastern islands of the archipelago (RAND 2014), yielding a sample that favours the relatively well-developed areas in western Indonesia. Alkire and Santos (2014) carried out further study on Indonesia using the Demographic Health Survey (ICF International 2012) data as a part of a grand endeavour to construct a globally comparable MPI (UNDP 2010). While they are completely representative of the population, the DHS data do not, however, provide household consumption expenditure information, preventing a useful comparison with the official measure of consumption poverty.

The contribution of the present study to the existing literature is threefold. Firstly, in estimating the extent of multidimensional poverty in Indonesia, this study uses large and nationally representative data that have been regarded as the primary source of information among Indonesian policy makers as well as international observers. While concurring with Alkire and Santos (2014: 266) who stress that data availability has been the major bottleneck in the development of an internationally comparable MPI, we would like to demonstrate that even when using an existing data source, the construction of an Indonesian MPI is not only technically feasible but also substantively meaningful. The collection of better well-being data is of course desirable, but Indonesians do not have to wait until the ‘perfect’ data becomes available to have their progress assessed. Secondly, the inclusion of consumption expenditure information makes this version of Indonesian MPI not only comparable to the official poverty measure, but also sensitive to economic fluctuations (Ravallion 2010: 11). Lastly, by providing an annual analysis of the trend of multidimensional poverty in the last 11 years, this study presents a richer picture compared to one that analyses only selected points in time over the same period.

The remainder of the paper is structured as follows. The next section describes the AF method, the data and the dimensions. It then investigates the degree to which income poverty correlates with non-income deprivations. Section 3 presents the results. Initially, unidimensional deprivations are investigated using the marginal dashboard approach. Then, MPI estimates at national and sub-national levels are presented along with robustness checks. Finally, changes in the distribution of deprivations among the poor are studied. Section 4 concludes.

## Methods

### The Alkire–Foster Method

This section describes the Alkire–Foster method for multidimensional poverty measurement (Alkire and Foster 2011a). For brevity, we focus only on those aspects of the methodology that are directly relevant to the present study. We also limit our attention to the general case, where the social indicators being considered might not have cardinal meaning. Further in-depth expositions are available in Alkire and Foster (2011a), Alkire and Foster (2011b), Alkire and Santos (2013) and Seth and Alkire (2014).

#### Setup

Before describing the identification and the aggregation steps of the Alkire–Foster method, it is necessary to outline some preliminary setups. First of all, let us consider an $n\times d$ dimensional achievement matrix $x=\left[ x_{ij}\right]$, in which the row $i=1,\ldots ,n$ indexes the individuals under study and the column $j=1,\ldots ,j$ indexes indicators for every dimension that the society cares about. No restriction is placed on the cardinality of indicators entered into the matrix; ordinal variables are acceptable. In this matrix, individuals’ achievements are recorded in the row vectors ($x_{i\cdot }$), while the marginal distribution of achievements is reflected in the column vectors ($x_{\cdot j}$). We also define a deprivation cut-off vector $z=(z_{1},\ldots ,z_{j})$ indicating the minimum level of achievement in every social indicator that should be attained by each individual in the society. The relative importance (trade-off) of each achievement indicator in the achievement matrix *x* is governed by a vector of weight $w=(w_{1},\ldots ,w_{d}$) such that $\sum _{j=1}^{d}w_{j}=d$. Of course, the choice of indicator, deprivation cut-off, and weighting scheme is largely contingent upon the specific context of study (what the society values, the aim and scope of the study, or data availability) and is open to public debate (Alkire 2011).

#### Identification

In the AF framework, identification begins with the construction of a deprivation matrix $g^{0}=\left[ g_{ij}^{0}\right]$ whose element is defined as $g_{ij}^{0}=w_{j}$ if $x_{ij}<z_{j}$ and $g_{ij}^{0}=0$ if otherwise. This deprivation matrix contains information about ‘who is deprived in which indicator and how much weight the indicators carry’ (Alkire and Santos 2013: 242). From $g^{0}$ matrix, a deprivation count vector $c=(c_{i},\ldots ,c_{n})$ whose element is $c_{i}=\sum _{j=1}^{d}g_{ij}^{0}$ is constructed. This column vector stores the sum of weighted deprivations experienced by each individual under study. The Alkire–Foster identification function $\rho _{k}(x_{i};z)$ is such that $\rho _{k}(x_{i};z)=1$ if $c_{i}\ge k$ and $\rho _{k}(x_{i};z)=0$ if otherwise, where *k* is the poverty cut-off denoting the minimum sum of weighted deprivations required to be multidimensionally poor (Alkire and Santos 2013). The plausible choice of poverty cut-off is $k\in \left[ min(w_{j}),d\right]$ and like other parameters in the AF framework, its value may be subjected to sensitivity analysis. Naturally, one would expect that the larger the *k*, the smaller the number of individuals identified as multidimensionally poor, and vice versa. When $k=min(w_{j})$, a *union* identification criterion is obtained, but when $k=d$, an *intersection* identification criterion is reached. In practice, however, an *intermediate* criterion (*k* = 0.33–0.50) is usually preferred (Alkire 2011).

Having assessed how deprived each individual is and identified who the poor are, the next step is to construct a censored deprivation matrix $g^{0}(k)=\left[ g_{ij}^{0}(k)\right]$ whose element is defined as $g_{ij}^{0}(k)=g_{ij}^{0}$ if $c_{i}\ge k$ and $g_{ij}^{0}(k)=0$ if otherwise. Likewise, a censored deprivation count vector is constructed such that $c_{i}(k)=c_{i}$ if $c_{i}\ge k$ and $c_{i}(k)=0$ if otherwise. This censoring mechanism allows analysts to focus only on those individuals who are identified as multidimensionally poor, guaranteeing that the aggregate poverty measure is insensitive to the achievement of non-poor individuals.

#### Aggregation

The main aggregation method in the AF family of multidimensional poverty measure is the *adjusted headcount ratio* or $M_{0}$, which is ‘the proportion of weighted deprivations that the poor experience in a society out of all the total potential deprivations that the society could experience’ (Santos 2013: 261). It is obtained by taking the arithmetic mean of the censored deprivation matrix $g^{0}(k)$:

1 $$M_{0}(x;z)= \frac{1}{nd}\sum _{i=1}^{n}\sum _{j=1}^{d}g_{ij}^{0}(k)$$

2 $$= \frac{1}{n}\underbrace{\sum _{i=1}^{n}\left[ \frac{1}{d} \sum _{j=1}^{d}g_{ij}^{0}(k) \right] }_{{\text {individual\,poverty}}}$$

3 $$= \frac{1}{d}\underbrace{\sum _{j=1}^{d} \left[ \frac{1}{n}\sum _{i=1}^{n}g_{ij}^{0}(k) \right] }_{{\text {censored\,deprivations}}}$$

4 $$= \underbrace{\frac{1}{n}q(k)}_{{\text {incidence (H)}}} \underbrace{\left[ \frac{1}{q(k)} \sum _{i=1}^{q(k)}\frac{c_{i}(k)}{d} \right] }_{{\text {intensity(A)}}}\quad {\text {where}}\quad q(k)= \underbrace{\sum _{i=1}^{n}\rho _{k}(x_{i};z)}_{{\text {head\,count}}}$$

Intuitively, $M_{0}$ can also be understood either as the weighted sum of individual poverty (Eq. 2), the weighted sum of censored deprivations by indicators (Eq. 3), or the intensity-adjusted poverty incidence (Eq. 4: $M_{0}=H\times A$). The measure is, as its name implies, simultaneously sensitive to both the prevalence (incidence) and the scope (average deprivation among the poor, or intensity) of poverty. By definition, it is expected that as *k* increases, *H* will get smaller and *A* will get larger, and vice versa.

#### Decomposition

Because the adjusted headcount ratio can be expressed as the weighted sum of individual poverty (Eq. 2), the measure is decomposable by population subgroups. It follows that overall poverty can be expressed as the weighted sum of poverty measures in *l* number of population subgroups:

5 $$M_{0}=\sum _{s=1}^{l}\frac{n_{s}}{n}M_{0}^{(s)}$$

and the contribution of a population subgroup *s* to the overall poverty $M_{0}$ is:

6 $$C_{s}=\frac{n_{s}}{n}\times \frac{M_{0}^{(s)}}{M_{0}} \quad {\text {for}}\quad s=1,\ldots ,l$$

where $\frac{n_{s}}{n}$ and $M_{0}^{(s)}$are the population share and the adjusted headcount ratio of subgroup *s*, respectively. Such a decomposition enables the assessment of the extent of inequality among subgroups by comparing each subgroup’s contribution to overall poverty relative to its population share (Alkire and Santos 2013: 245). A severe deviation from $C_{s}/\left( \frac{n_{s}}{n}\right) =1$ is indicative of the fact that a particular subgroup bears a disproportionately large (or small) share of poverty.

Similarly, the adjusted headcount ratio can also be broken down by its indicators, because the measure is expressible as the weighted sum of the censored deprivations by indicators (Eq. 3). The overall poverty can thus be expressed as:

7 $$M_{0}=\sum _{j=1}^{d}\left( \frac{w_{j}}{d}\right) h_{j}(k)$$

and the contribution of a social indicator *j* to overall poverty $M_{0}$ is:

8 $$C_{j}=\frac{w_{j}}{d}\times \frac{h_{j}(k)}{M_{0}}\quad {\text {for}}\quad j=1,\ldots ,d$$

where $h_{j}(k)$ is the censored headcount ratio of indicator *j*. From this, we know that whenever $C_{j}/\left( \frac{w_{j}}{d}\right)$ deviates severely from unity, then there is a relatively high (or low) deprivation in an indicator (Alkire and Santos 2013: 245). Dimensional contribution is obtainable simply by adding up $C_{j}$ within a particular dimension.

#### Robustness Analysis

In the AF framework, robustness is established through sensitivity analysis employing different sets of indicators, deprivation cut-off, weight, or poverty cut-off (Alkire and Santos 2014). In this study, we apply poverty cut-off dominance analysis, confidence intervals overlap testing, and rank correlation testing, which constitute the standard robustness toolbox for the AF family of multidimensional poverty measures.

#### Rate of Change

Once some degree of robustness has been established, the rate of inter-temporal change in aggregate poverty can be calculated (Alkire and Vaz 2014). The absolute $(\Delta M_{0})$ and relative $(\delta M_{0})$ rates of change are defined as follows:

9 $$\Delta M_{0}= M_{0}^{(t2)}-M_{0}^{(t1)}$$

10 $$\delta M_{0}= \frac{M_{0}^{(t2)}-M_{0}^{(t1)}}{M_{0}^{(t1)}}\times 100$$

where *t*2 and *t*1 denote the later and the initial time points, respectively. When the two time points span over a number of years, it is sometimes useful to express the changes in their annualised values:

11 $$\bar{\Delta }M_{0}= \frac{M_{0}^{(t2)}-M_{0}^{(t1)}}{t2-t1}$$

12 $$\bar{\delta }M_{0}= \left[ \left( \frac{M_{0}^{(t2)}}{M_{0}^{(t1)}}\right) ^{\frac{1}{t2-t1}} -1\right] \times 100$$

which give us the average absolute (or relative) change during the period of observation.

#### Inequality Among the Poor and Across Subgroups

Finally, after assessing the incidence and intensity of multidimensional poverty, it is only natural to ask whether poverty reduction, if any, has been inclusive among the poor and uniform across population subgroups (the ‘triple I’ of poverty: incidence, intensity, inequality; Sen 1976). Finding the right inequality measure for such a purpose has been proven to be non-trivial in the multidimensional setting; Seth and Alkire (2014) recently proposed a decomposable inequality measure based on the *positive-multiple of variance* to overcome the obstacles stemming mainly from the use of non-cardinal indicator variables in the construction of $M_{0}$. Following their proposal, inequality (*I*) among poor individuals $(I^{q})$ and across subgroups $(I^{s})$ can be expressed as:

13 $$I^{q}= \tilde{\beta }\times \frac{1}{q(k)}\sum _{i=1}^{q(k)} \left[ c_{i}(k)-A\right] ^{2}$$

14 $$I^{s}= \tilde{\beta }\times \sum _{s=1}^{l}\frac{n^{s}}{n} \left[ M_{0}^{(s)}-M_{0}\right] ^{2}$$

where $\tilde{\beta }$ is a normalisation factor that must be chosen such that $I=\left[ 0,1\right]$, respecting the properties of any standard inequality index. Because it is known that ‘the maximum possible value that variance takes is one fourth of the range of the deprivation score vector, which is attained when half of the population have the lowest deprivation scores and the other half have the highest deprivation scores’ (Seth and Alkire 2014: 16), $\tilde{\beta }$ in the between-poor equation equals the inverse of $\frac{1}{4}\left\{ max\left[ c_{i}(k)\right] -min\left[ c_{i}(k)\right] \right\} ^{2}$. Accordingly, as $M_{0}=\left[ 0,1\right]$ then it is obvious that $\tilde{\beta }=4$ in the between-subgroup equation.

### Data

We analyse data from the *Survei Sosial Ekonomi Nasional* (National Socio-economic Survey; henceforth Susenas), an annual cross-sectional household survey administered by the Indonesian Statistical Bureau (*Badan Pusat Statistik*, BPS). Initiated in 1963, Susenas is a large and nationally representative survey, which has for decades served as the main source of information not only for the government of Indonesia but also for many international bodies, including the World Bank PovcalNet (see also Surbakti 1995; van de Walle 1988). The survey consists of a yearly core module (health, education, employment, household consumption expenditure, housing, fertility, contraception, and communication) and one of three alternating modules on (1) culture and education, (2) housing and health, and (3) household consumption expenditure, each administered once every three years.

Compared to other Indonesian household survey data available to date, the strength of Susenas lies in (1) its comprehensive information on consumption expenditure (more than 300 food and non-food items in 2013), education and literacy; and in (2) its large sample and periodicity which permit precise annual inferences to be made at low levels of administration. However, it should be noted that the information on health available in Susenas is neither as comprehensive as that available in the Demographic and Health Survey (DHS) nor in the Indonesia Family Life Survey (IFLS) that were analysed previously by Alkire and Santos (2014) and Alkire and Foster (2011a). While Susenas records information on the number of disabled days and morbidity for each individual, it does not provide any anthropometric measure. Of course, a household survey that was first designed nearly four decades ago is by no means ideal for the contemporary purpose of multidimensional poverty measurement, but aside from this, the consumption expenditure data available in Susenas provide us with the opportunity to address the concern about the unresponsiveness to economic fluctuations (Ravallion 2010: 11) of the living standard indicators used in the current version of UNDP’s multidimensional poverty index (Alkire and Santos 2014; UNDP 2010).

Since the Susenas sample is drawn using a multi-stage stratified random sampling design (urban/rural stratification, census blocks as the primary sampling unit, households within each block as the secondary sampling unit), the survey design along with the sampling weight is always incorporated into analysis. Our exploration indicates that ignoring the unequal sampling probability underestimates the proportion of individuals living on Java island severely (30 % instead of 60 %), leading to a potentially biased estimate of the population parameter.

We analyse eleven consecutive years of Susenas data, from 2003 (just before the enactment of Law 32/2004 on Regional Government that marked the decentralisation era; 346 districts) to 2013 (the latest available Susenas; 499 districts). We regroup all districts that split during the period of observation into their original 2003 districts. The unit of analysis is an individual aged 18 and older. Children are excluded from the analysis because the relevant dimensions of their well-being depend on their age (Roche 2013), and information on those dimensions are missing from Susenas. We believe that children deserve special consideration that takes into account their own specificities (see Trani et al. 2013 and the cited works therein). Only complete cases are used in the analysis: individuals that have any social indicator (presented next) missing are dropped from the sample. This yields a total complete-case sample size of 7,148,964 individuals ($N\approx 650{,}000$ per year) with each survey wave having a final sample size of 91–100 % of the original sample size.

### Dimensions, Indicators, Cut-Offs and Weights

In an ideal world, the choice of dimensions, indicators, cut-offs and weights for the measurement of multidimensional poverty would be guided by the revealed preferences of the poor (what the poor think of being poor, what deprivations matter the most, and what trade-offs the poor assign between deprivations). Yet, with the notable exceptions of Mexico’s *Voices of the Poor* study (Székely 2003) and Bhutan’s *Gross National Happiness* survey (Santos and Ura 2008; Ura et al. 2012), large-scale participatory exercises are rare. In contrast to these countries, the official conceptualisation of poverty in Indonesia is still the traditional consumption (or income) poverty measurement, defined as the failure to attain the consumption level required for the fulfilment of a basket of basic food and non-food needs (BPS 2015c). The idea of poverty as an experience of simultaneous deprivations has rarely penetrated the nation’s discourse of development (for example see Hill 2014 or Strauss et al. 2004). In this light, we base our elicitation of dimensions, indicators, cut-offs and weights on the existing Human Development Index (HDI; UNDP 2010), multidimensional poverty index (MPI; UNDP 2010), Millennium Development Goals (MDGs), and the 1945 Constitution of the Republic of Indonesia (MPR RI 2011), subject to constraints imposed by data availability in Susenas survey.

**Table 1:** Dimensions, indicators, deprivation cut-offs and relative weights

Dimension	Indicator variable	Deprivation cut-off ‘An individual is deprived if...’	Weight		
			*w*1	*w*2	*w*3
Income$$^{\mathrm{a}}$$	Per capita daily consumption	<$1.51 PPP	$$\frac{1}{3}$$	$$\frac{1}{3}$$	$$\frac{1}{3}$$
Health$$^{\mathrm{b}}$$	Illness episode	>4 days	$$\frac{1}{6}$$	$$\frac{1}{6}$$	$$\frac{1}{3}$$
	Morbidity	>3 diseases	$$\frac{1}{6}$$	$$\frac{1}{6}$$	0
Education$$^{\mathrm{c}}$$	Schooling	Has not completed primary school	$$\frac{1}{6}$$	$$\frac{1}{3}$$	$$\frac{1}{3}$$
	Literacy	Cannot read and write Latin characters	$$\frac{1}{6}$$	0	0

^a^The first MDGs. The fourth paragraph of preamble, article 27(2) and 28C(1) of the Constitution

^b^The fourth, fifth and sixth MDGs. Article 28H(1) and 34(3) of the Constitution

^c^The second MDGs. The fourth paragraph of preamble, article 28C(1), 31(1) and 31(2) of the Constitution

Three dimensions are included in our version of Indonesian MPI: (1) income, (2) health and (3) education, mimicking the UNDP’s latest HDI and MPI. Alkire and Santos (2014: 253) note that these dimensions are not only instrumental to many other vital outcomes but also intrinsically valuable in themselves. Furthermore, they argue that having only three dimensions simplifies communication and interpretability because ‘the contribution of the chosen dimensions is widely recognized across political and ideological divides’. As shown in Table 1, these dimensions are clearly related to the values of the Constitution, not to mention the MDGs.

Income is operationalised using per capita daily consumption, which is obtained by deflating total daily household expenditure by household size. The figure is measured in international dollar (an expression of purchasing power parity, or PPP; UNSD 2014) and adjusted for spatial cost-of-living differences using the provincial urban-rural adjustment factors derived from the relative differences between the national and the local poverty lines (Ilmma and Wai-Poi 2014: 132). Fixed adjustment factors (from 2008) are used for the entire 2003–2013 series because these data are not available prior to 2007 (BPS 2015a; see also Alkire et al. 2013: 2–4 for a similar approximation method). We consider an individual to be deprived in the income domain if his or her daily consumption is less than the Asia-specific poverty line of $1.51 (ADB 2014). This cut-off is more stringent than both Indonesia’s national poverty line ($1.43) and the World Bank’s extreme poverty line ($1.25).

Health status is assessed using two indicators: illness episode (number of days disabled within the last month) and morbidity (number of illnesses within the last month), which, admittedly, may not be as informative as the body mass index (BMI) indicator used in Alkire and Foster (2011a) and in Alkire and Santos (2014). However, these are the best available health measures in the Susenas survey, and since the disabling burden of poor health may lead not only to potentially missed income-generating opportunities (Schultz and Tansel 1997) but also, ultimately, to an unfulfilled life, we consider that these indicators make a good representation of the health domain. The inclusion of the illness episode indicator variable into a multidimensional poverty index is not new; such a measure has been used previously in a version of Bhutanese MPI (Santos 2013). In addition, the measure is often employed to operationalise Grossman’s model of health production function (Grossman 1972) in the health economics literature. An individual is deprived in health if he or she was ill for more than 4 days or caught more than 3 diseases within the last month (Table 1). These, we believe, are reasonable cut-offs considering the high prevalence (60–70 %) of informal self- and seasonal employment in Indonesia (Nazara 2010).

Like health, education is also operationalised using two indicators: the completion of primary school (schooling) and the ability to read and write Latin characters (literacy). An individual is deprived if he or she has not completed primary education or is illiterate (Table 1). Relative to other indicators described above, primary schooling and literacy are perhaps the most universally accepted social indicators. They are highly valued worldwide: their presence in the HDI, MPI, MDGs and even the 1945 Constitution of the Republic of Indonesia testifies to this.

Having chosen the social indicators to be included in the multidimensional poverty index, we now define their weights, which are necessary for identification purposes (Alkire 2011: 14–16). This weight assignment, which means making the trade-offs between social indicators explicit, clearly entails value judgements (Decancq and Lugo 2013). In this study, as in many other applications of the Alkire–Foster method, we use a normative weight because of the unavailability of preferences data. For the proposed Indonesian MPI, an equal-nested weighting scheme, which assigns an equal relative weight $(\frac{1}{3})$ to each dimension and also an equal weight to all indicators within a dimension, is used (weight *w*1 in Table 1). We then set the poverty cut-off to $k=\frac{1}{3}$ so that an income-poor individual is ‘automatically’ multidimensionally poor, but not the converse. This choice of parameters reflects the beliefs that (1) income, health and education are equally important for human development and (2) income still holds a special position in poverty measurement ‘given its fungibility and its key role in facilitating other capabilities’ (Foster 2007: 9). Notwithstanding the preference for this setting, we still conduct sensitivity analysis employing alternative weighting schemes (weights *w*2 and *w*3 in Table 1) and/or poverty cut-offs for $k\in \left[ \frac{1}{6},1\right]$.

**Table 2:** Spearman correlation matrix of deprivations (2003–2013 maximum value; unweighted sample)

	Income	Illness episode	Morbidity	Schooling	Literacy
Income	1.00				
Illness episode	0.02	1.00			
Morbidity	0.01	0.23	1.00		
Schooling	0.14	0.10	0.06	1.00	
Literacy	0.13	0.10	0.05	0.34	1.00

At this point, critics may contend that an index of multidimensional poverty is unnecessary because the social indicators included in its construct are, presumably, highly correlated either to income or to each other, representing a ‘double counting’. Our Indonesian data prove that this is not the case. As shown in Table 2, the correlation between income poverty and other deprivations in health and education during the last decade is never larger than 0.14; the figure among indicators of health and education is always less than 0.35. This mismatch between income poverty and deprivations in other social indicators conforms to the general finding in the literature (see Battison et al. 2013 on Latin American countries; Batana 2013 and Klasen 2000 on Africa; Brandolini and D’Alessio 1998 on Italy; Ranis and Stewart 2012 on Bangladesh, Chile, Indonesia, Kazakhstan, Laos and Zambia; Santos 2013 on Bhutan; Whelan et al. 2004 on Europe; Yu 2013 on China), providing ‘good empirical basis to support a multidimensional approach to poverty measurement, which goes beyond income and asset ownership’ (Santos 2013: 267).

## Results

### Unidimensional deprivations

**Fig. 1:**
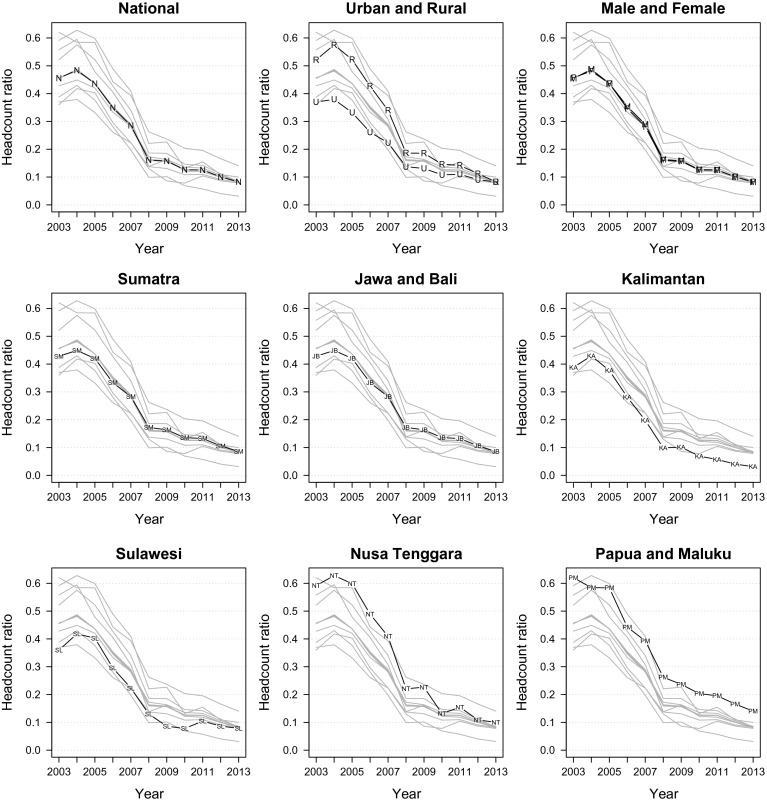
Income poverty

We begin by describing the trend of unidimensional deprivations in Indonesia during the 2003–2013 period. The top-left panel of Fig. 1 shows a strong 83 % income poverty reduction at the national level. Nearly half (46 %) of adult Indonesians were income poor in 2003, but a decade later, the figure improved significantly to just 8 %, registering an absolute 0.38 point reduction. It is apparent from the trend-line that income poverty reduction is characterised by two quinquennial regimes. Reduction was faster in the 2003–2008 period (65 %) than in the 2008–2013 period (50 %), a phenomenon that is consistent with the fact that the global economy was relatively more buoyant in the former period (high real growth rate and high commodity prices; Bourguignon et al. 2008: 12–13) than in the latter (the 2008 financial crisis and the ensuing drop in commodity prices; Battison et al. 2013: 308). At the same time, this discontinuity could reflect the differing efficacy in governance between the first (2004–2009) and the second United Indonesia Cabinets (2009–2014).

The top-middle panel of Fig. 1 shows that income poverty reduction has been accompanied by a substantial improvement in the urban/rural disparity. The rural-to-urban poverty ratio fell sharply from 1.41 in 2003 to only 1.01 in 2013. The gender gap had not been of serious concern over the 11 years of observation, as the female-to-male poverty ratio has hardly ever deviated from the 1.00–1.01 range (the top-right panel of Fig. 1). However, we should keep in mind that this figure is obtained from household expenditure data rather than from individual income data. The trend in regional disparity seems to be similar to that of the urban/rural one. When we analyse each island-group separately (the middle to bottom panels of Fig. 1), the pattern of two-regime poverty reduction pattern still holds, while the between-island variance shrank by 90 % from 0.013 in 2003 to 0.001 in 2013, indicating a converging regional poverty. Despite this tremendous progress, it should be noted that Papua and Maluku, whose headcount ratio has remained constant at 1.5 times higher than the national average since 2008, seem to be left behind. Moreover, it is noticeable that Sulawesi has failed to register any significant improvement after 2008. This suggests that while Indonesia has enjoyed substantial progress in terms of sharply reduced income poverty and gradually converging urban/rural as well as regional disparities during the last decade, the East-West divide remains (see also poverty maps in Fig. 7).

**Fig. 2:**
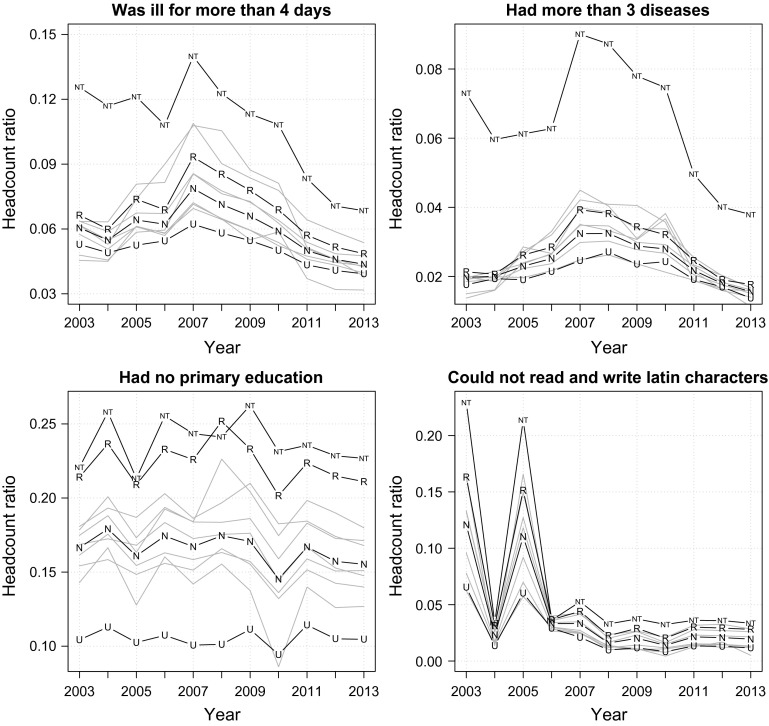
Non-income deprivations

Having described the state of income poverty, we now investigate trends in health and education (Fig. 2). Since non-income achievements are usually represented by stock rather than flow variables, and are therefore unlikely to change in the short run (Battison et al. 2013: 296), it is expected that their trend-lines will be relatively more stable than that of income.

Some clear patterns emerge from the two plots in the top panel of Fig. 2, which displays the evolution of health deprivations over the decade. First, while the reduction of income poverty was at its fastest rate (2003–2008), the nation’s illness episode deprivation increased by 7 % year-on-year (13 % for morbidity) before eventually peaking in 2007 and then gradually returning to its initial level in 2010/2011. This inverted U-shape trend-line suggests that there may have been a short-term surge of negative health-related behaviour that followed the rising income level. In addition, it might also capture the health cost of both natural and man-made disasters (the Indian ocean earthquake and tsunami, the Java earthquake, the Sumatra flood and earthquake, the Sulawesi flood and landslide, and the Sidoarjo mud flow, to name only a few) that occurred relentlessly during the 2004–2007 period. Second, the trajectory of rural-to-urban health deprivation ratio also follows this inverted-U shape. The figure was about 1.20, 1.50 and 1.20 in 2003, 2007 and 2013, respectively, suggesting little to no improvement in terms of urban/rural health equality. The plots also present evidence regarding the disturbingly weak health status in Nusa Tenggara islands. Illness episode deprivation in Nusa Tenggara was 1.53–2.13 times greater than the national average and the figure for morbidity was in the range of 2.23–3.70 times greater. Moreover, it is evident that Nusa Tenggara exhibits a very distinctive trend-line compared to the rest of Indonesia, thereby exerting undue influence over the between-island variability. In contrast to the patterns of urban/rural and regional disparities, the female-to-male health deprivation ratio has always been stable in the range of 0.80–0.90, indicating that Indonesian women seem to be slightly healthier than their male counterparts (trend-lines greyed out).

The two plots at the bottom panel of Fig. 2 show the trends of schooling and literacy deprivations. With the exception of two irregularly spiking literacy deprivations in 2003 and 2005, which appear to be a data quality problem, the trends seem to be stable over the window of observation. The rural-to-urban education deprivation ratio was constant in the range of 2.00–2.25 for both indicators. The female-to-male deprivation ratio was at about 1.20 for schooling and 1.80 for literacy; and the between-island variability has barely changed over the decade. It is evident that nearly a quarter of Indonesian adults living in rural areas failed to complete primary school, despite the substantial reduction in income poverty and the constitutional mandate for the provision of universal primary schooling. Again, by studying all four plots shown in Fig. 2, one can see immidiately that Nusa Tenggara islands are doubly burdened by both poor health and education outcomes.

These findings demonstrate that Indonesia’s laudatory income poverty reduction over the last decade has not been complemented by equivalently strong improvements in non-income dimensions. The findings also suggest that different population subgroups (urban/rural, men/women, island-groups) performed differently in different dimensions of well-being.

We now ask some follow-up questions. Taking those social indicators altogether, how many Indonesians are poor overall? Are urban areas always better off? Which island within the archipelago is the most deprived? What happened to gender and spatial inequalities? The marginal dashboard approach (Ravallion 2010, 2011) that we have just applied throughout this section is incapable of answering these questions because it only allows us to look at the *marginal distribution* of deprivations, while our inquiries demand a characterisation of the *joint distribution* of multiple deprivations (Alkire et al. 2011). In other words, in order to be able to answer these questions, the poverty measure has to take into account the extent of simultaneous deprivation experienced by individuals in the society. The multidimensional poverty measure to be reported next does just that.

### Multidimensional Poverty at the National Level

**Fig. 3:**
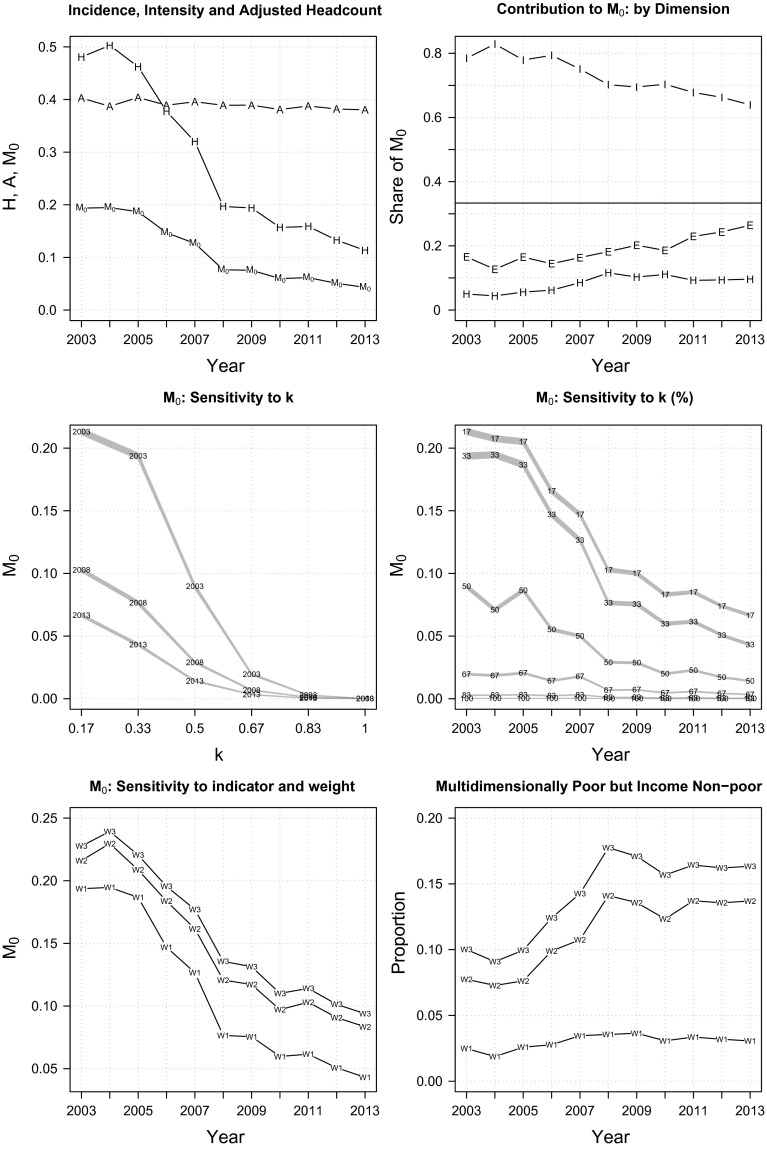
Multidimensional poverty at the national level

Figure 3 presents the trend of multidimensional poverty at the national level. The top-left panel shows that overall poverty $(M_{0})$ has declined at an annual rate of 14 % over the 2003–2013 period, owing much to the sharp reduction in the proportion of individuals identified as multidimensionally poor (poverty incidence, *H*), but less to the improvement in the average deprivations experienced by the poor (poverty intensity, *A*). In 2003, 48 % of Indonesian adults were multidimensionally poor and, collectively, they experienced about one-fifth (0.19) of the total possible deprivations that all adults could experience. A decade later, only 11 % of adults were poor: the overall poverty figure was just 0.04, indicating a substantial 78 % improvement. As in the case of income poverty, reduction in multidimensional poverty was also faster in the first six years $(\delta M_{0}=60\,\%)$ than in the second five $(\delta M_{0}=44\,\%)$.

Despite of a steady 2 % annual decline in the contribution to overall poverty, income remains the main contributor to multidimensional poverty (the top-right panel of Fig. 3). Its contribution to overall poverty in 2013 was still about two times larger than its relative weight (64 %). On the other hand, health and education contributed less than their relative weights, suggesting that there were relatively low deprivations in these dimensions. It is noteworthy, however, that as time passes, the contribution of non-income dimensions to overall poverty increases steadily.

**Table 3:** Correlation matrix of rank orderings across different weights

Ranking pair^†^	2003	2004	2005	2006	2007	2008	2009	2010	2011	2012	2013
*National*											
$\tau _{B}(w1,w2)$	1*										
$\tau _{B}(w1,w3)$	1*										
$\tau _{B}(w2,w3)$	1*										
*Urban/rural*											
$\tau _{B}(w1,w2)$	1	1	1	1	1	1	1	1	1	1	1
$\tau _{B}(w1,w3)$	1	1	1	1	1	1	1	1	1	1	1
$\tau _{B}(w2,w3)$	1	1	1	1	1	1	1	1	1	1	1
*Gender*											
$\tau _{B}(w1,w2)$	1	1	1	1	1	−1	1	1	1	1	1
$\tau _{B}(w1,w3)$	1	1	1	1	1	−1	1	1	1	1	1
$\tau _{B}(w2,w3)$	1	1	1	1	1	1	1	1	1	1	1
*Island*											
$\tau _{B}(w1,w2)$	1	0.87	1	0.87	1	0.87	0.73	0.87	0.73	0.73	1
$\tau _{B}(w1,w3)$	1	1	0.87	0.87	0.87	0.87	0.87	1	0.47	0.73	0.87
$\tau _{B}(w2,w3)$	1	0.87	0.87	1	0.87	1	0.87	0.87	0.73	1	0.87

$^\dagger$ Reported are Kendall’s $\tau _B$ correlation coefficient for pairs of ranking while holding $k=\frac{1}{3}$

* Correlation coefficient for pairs of year ranking at the national level (2003–2013)

The middle panel of Fig. 3 displays the results of poverty cut-off dominance analysis. On the left, we plot the estimated adjusted headcount ratio $(M_{0})$ for the year 2003, 2008 and 2013, along with their analytical 95 % confidence intervals, against all possible poverty cut-offs spanning from the union $(k=\frac{1}{6})$ to the intersection $(k=1)$ identification criterion. It turns out that the curves never cross, nor do their confidence intervals overlap, meaning that there was an unambiguous poverty reduction over the 2003–2013 period. On the right, we present similar analysis, plotting the $M_{0}$ estimates for each poverty cut-off against the year. Results suggest that the shape of the trend-line is robust to any poverty cut-off for $k\in \left[ \frac{1}{6},\frac{1}{2}\right]$. Furthermore, the data reveal that even when alternative weights are specified, the shape of the downward-sloping trend (the bottom-left panel of Fig. 3) as well as the ordering of year ranking (the first five rows of Table 3) remain largely unaltered.

In the bottom-right panel of Fig. 3, we calculate the extent of mismatch when targeting the poor using the conventional measure of income poverty versus using the proposed multidimensional poverty index $(k=\frac{1}{3})$. We found that the mismatch is about 3 % for the baseline weight (*w*1), which equals approximately 4.5 million adult Indonesians in 2013. The discrepancy increases significantly as we specified alternative weights that assign more importance to the schooling indicator (*w*2; 7–14 %), or to both schooling and illness episode indicators (*w*3; 9–17 %). This finding, in combination with that of the steadily increasing contribution of non-income deprivations to overall poverty (the top-right panel of Fig. 3), underlines the growing relevance of the multidimensional conceptualisation of poverty in Indonesia.

### Multidimensional Poverty Across Population Subgroups

Having described the trend of multidimensional poverty at the national level, we now decompose the national MPI into contextually relevant subgroups, which, in Indonesia, means measuring poverty by urban/rural, gender and island-group separately. The goals of this exercise are to understand whether the pattern of poverty reduction has been uniform across subgroups and to identify any especially disadvantaged segment of Indonesian society.

**Fig. 4:**
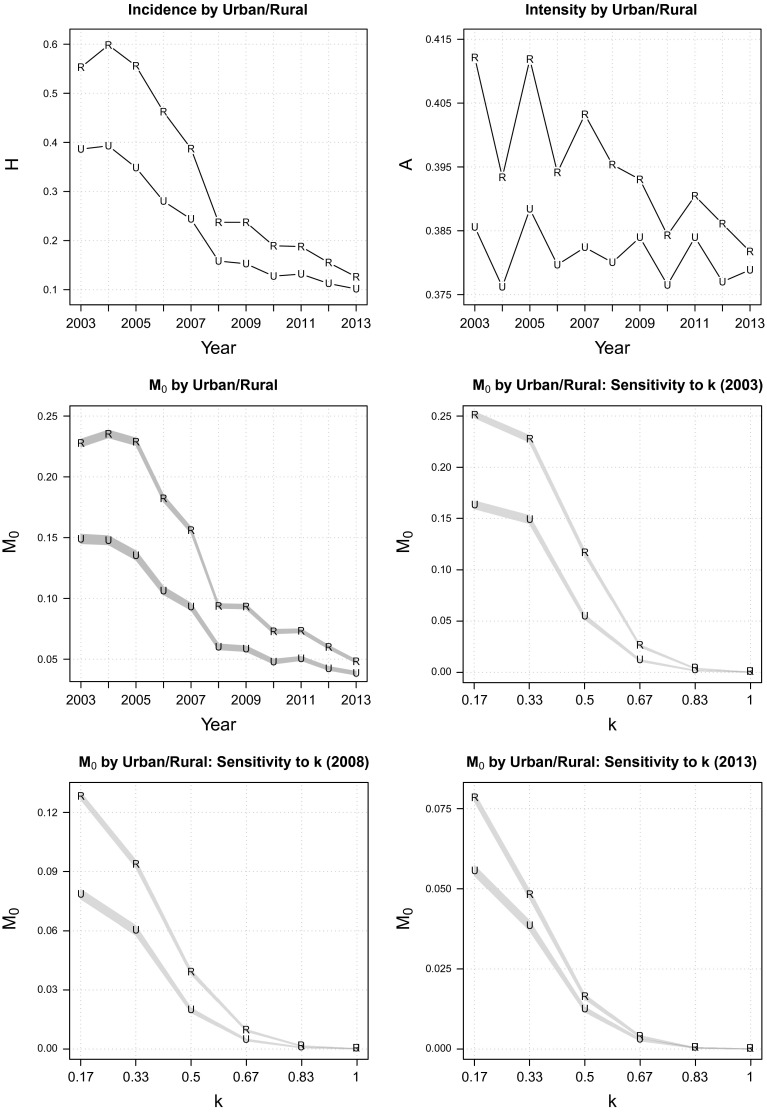
Multidimensional poverty by urban/rural

Figure 4 displays the trend of urban/rural poverty. In both urban and rural areas, multidimensional poverty has declined significantly over the 11 years of observation (the middle-left panel of Fig. 4). Progress was faster in rural than in urban areas $(\delta M_{0}=79$ vs. 74 %), resulting in a progressively narrowing rural-to-urban poverty ratio (1.53 in 2003 vs. 1.25 in 2013). Two distinct patterns emerge as to how poverty reduction was achieved. As shown in the top panel of Fig. 4, poverty reduction in rural areas was driven by improvement in *both* poverty incidence $(\delta H=77\,\%)$ and intensity $(\delta A=7.4\,\%)$ whereas in urban areas, where amelioration in intensity was minuscule $(\delta A=1.7\,\%)$, poverty reduction was chiefly attributable to the diminishing proportion of individuals experiencing multiple deprivations $(\delta H=74\,\%)$. Results of poverty cut-off dominance analysis (the bottom four plots in Fig. 4) and a rank correlation test (Table 3) suggest that multidimensional poverty was unambiguously higher in rural than in urban areas for *each* year in the 2003–2013 period.

**Fig. 5:**
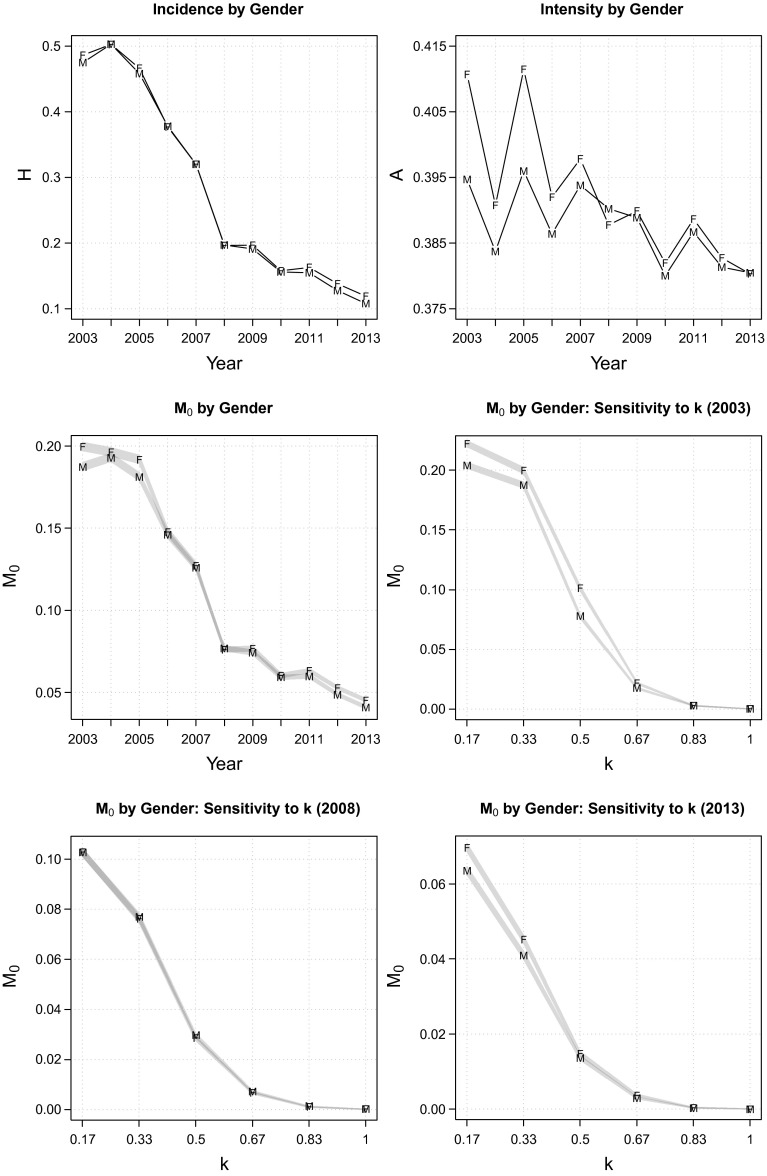
Multidimensional poverty by gender

Next, we examine multidimensional poverty by gender. The data reveal that the female-to-male poverty ratio has never oscillated outside the 0.99 (2008) to 1.10 (2013) range (the middle-left panel of Fig. 5). In fact, the proportion of Indonesian women experiencing simultaneous deprivations over the last decade did not differ much compared to that of their male counterparts (the top-left panel of Fig. 5). Poverty intensity was slightly higher (1–4 %) among women than men during the 2003–2007 period, but since 2008, there has been hardly any difference with regard to the average deprivations experienced by the poor of both genders (the top-right panel of Fig. 5). A rank correlation test in Table 3 indicates that the gender ranking is generally robust to the choice of weight, but the results of poverty cut-off dominance analysis presented in the middle and bottom panels of Fig. 5 suggest that women are not unambiguously more deprived than men. This result, however, should be interpreted with caution because we still cannot disentangle the precise income (consumption) of men and women that live in the same household using household expenditure data.

**Fig. 6:**
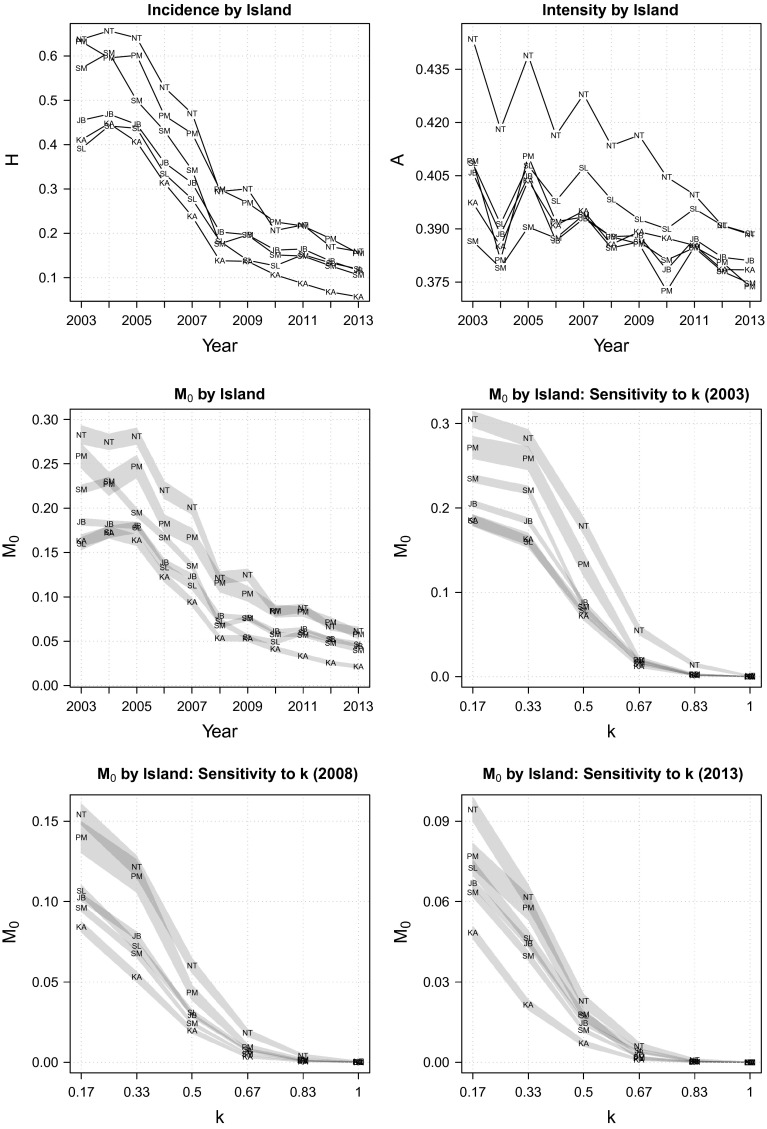
Multidimensional poverty by island

We now turn to investigating the trend of regional poverty (Fig. 6). In general, the last decade saw a substantial poverty reduction in all six island-groups that make up the Indonesian archipelago (the middle-left panel of Fig. 6). With the exception of Nusa Tenggara islands, improvement was principally attributable to diminishing poverty incidence, with only minimal progress in terms of poverty intensity (the top panel of Fig. 6). Poverty reduction was faster in the 2003–2008 period ($\delta M_{0}$ = 55–67 %) than in 2008–2013 ($\delta M_{0}$ = 36–60 %). The fastest progress was observed in Kalimantan $(\bar{\delta }M_{0}=18\,\%)$, whereas the slowest was in Sulawesi $(\bar{\delta }M_{0}=12\,\%)$.

Robustness tests for the between-island comparison fail to yield clear-cut results. On the one hand, the rank correlation test in Table 3 shows that the ranking of regional poverty is relatively robust to the selection of weight. On the other hand, poverty cut-off dominance analysis presented in the middle to the bottom panels of Fig. 6 reveals that there are only limited dominances between the islands *and* over the 11-year period. Yet, within the limited scope of statistically meaningful comparisons that can be drawn, we can still at least deduce that from 2010 onwards, Papua and Maluku, along with Nusa Tenggara, have always been the poorest islands of the country, whereas Kalimantan is the least poor; between these two extremes are Sumatra, Java, Bali and Sulawesi, whose level of multidimensional poverty has always been close to the national average.

**Fig. 7:**
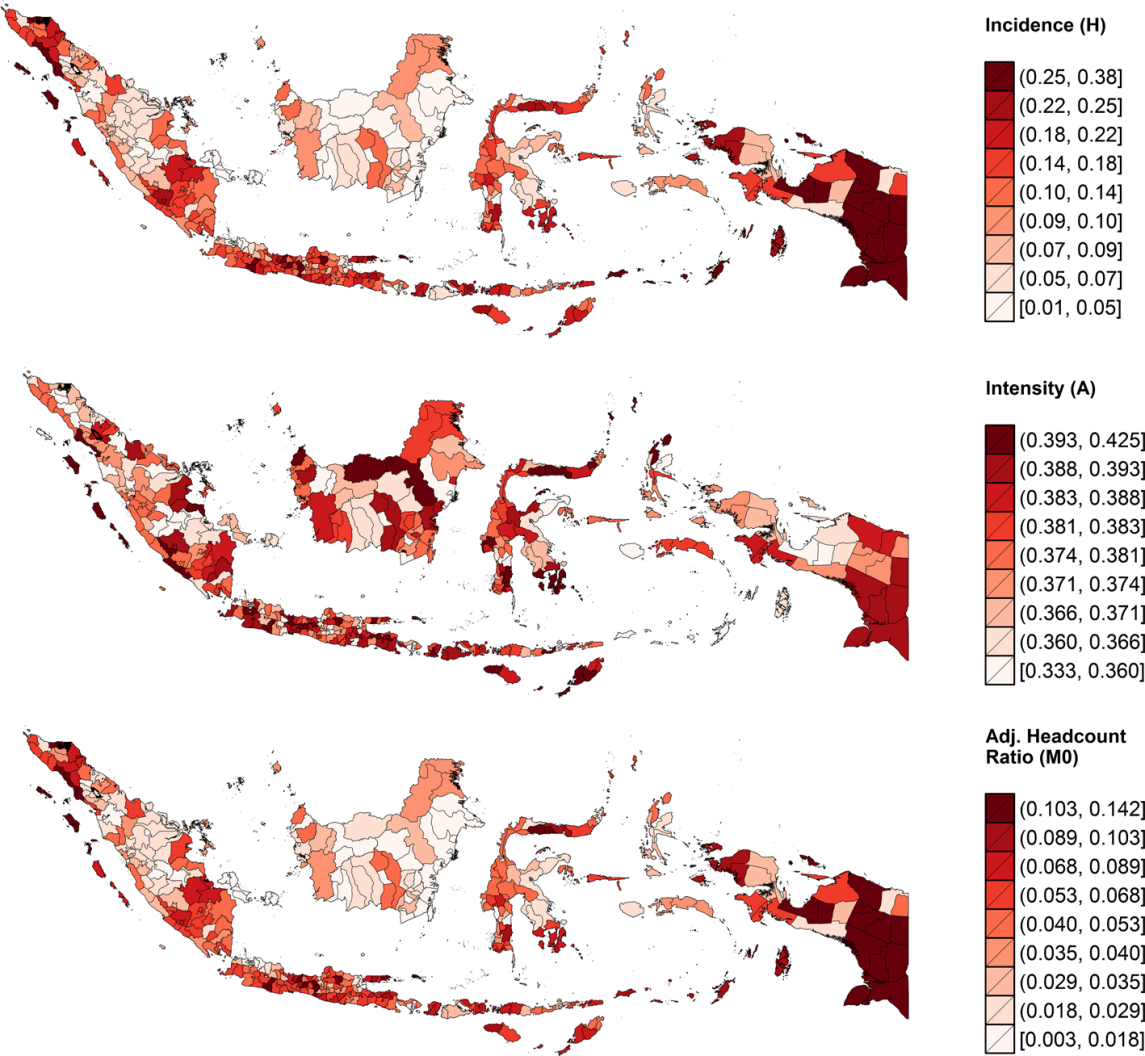
Incidence, intensity and adjusted headcount ratio at the district level (2013)

**Fig. 8:**
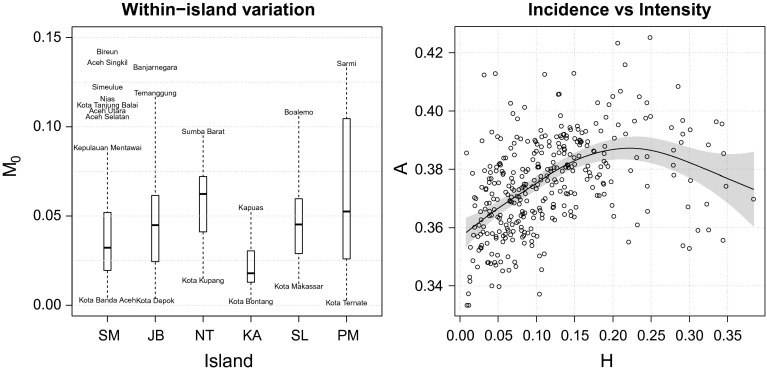
Within-island variation and districts’ incidence and intensity (2013)

**Table 4:** The top 10 poorest and the least poor districts in 2013

Incidence			Intensity			Adjusted Headcount Ratio		
Island	District	*H*	Island	District	*A*	Island	District	$M_{0}$
SM	Bireun	0.384	SL	Boalemo	0.425	SM	Bireuen	0.142
PM	Jayawijaya	0.348	SL	Bantaeng	0.423	SM	Aceh Singkil	0.136
PM	Sarmi	0.345	SL	Gorontalo	0.416	PM	Sarmi	0.133
SM	Simeulue	0.344	SL	Kepulauan Sangihe	0.413	JB	Banjarnegara	0.133
SM	Aceh Singkil	0.343	KA	Kapuas Hulu	0.413	PM	Jayawijaya	0.133
JB	Banjarnegara	0.335	KA	Sambas	0.413	PM	Pegunungan Bintang	0.126
PM	Paniai	0.325	KA	Kota Banjar Baru	0.412	SM	Simeulue	0.122
PM	Pegunungan Bintang	0.325	JB	Blora	0.409	PM	Biak Numfor	0.119
PM	Biak Numfor	0.323	SM	Kepulauan Mentawai	0.407	JB	Temanggung	0.199
SM	Kota Tanjung Balai	0.311	PM	Halmahera Utara	0.406	PM	Paniai	0.117
SM	Karo	0.018	JB	Kota Yogyakarta	0.345	SM	Karo	0.006
JB	Badung	0.017	PM	Kota Sorong	0.345	JB	Sidoarjo	0.006
JB	Sidoarjo	0.016	KA	Kota Samarinda	0.343	JB	Badung	0.006
JB	Kota Denpasar	0.013	SM	Kota Banda Aceh	0.342	JB	Kota Denpasar	0.005
SM	Kota Banda Aceh	0.013	SM	Kota Sabang	0.340	SM	Kota Banda Aceh	0.004
KA	Kota Samarinda	0.013	SM	Kota Pematang Siantar	0.340	KA	Kota Samarinda	0.004
KA	Kota Balikpapan	0.011	JB	Kota Depok	0.337	KA	Kota Balikpapan	0.004
JB	Kota Depok	0.011	SM	Kota Tebing Tinggi	0.337	JB	Kota Depok	0.004
PM	Kota Ternate	0.009	PM	Kota Ternate	0.333	KA	Kota Bontang	0.003
KA	Kota Bontang	0.008	KA	Kota Balikpapan	0.333	PM	Kota Ternate	0.003

Now, what happens if multidimensional poverty is measured at the lowest level of autonomous administrative areas instead of across island-groups? Figure 7 presents poverty maps displaying the extent of incidence, intensity, and overall poverty in 346 districts in Indonesia for the year 2013. It appears that the island ranking observed above still holds generally, but such comparison tends to conceal a large amount of variation between the districts on those islands. As we dig deeper (Table 4), it turns out that while five out of the ten poorest districts in 2013 are indeed located in Papua, three of them are in Sumatra (Aceh) and two are, perhaps surprisingly, in Central Java. There is unmistakable within-island variation as well (the left panel of Fig. 8). The island of Java, for example, is paradoxically home to one of the most (Banjarnegara, $M_{0}=0.133$) *and* the least (Kota Depok, $M_{0}=0.004$) deprived districts in Indonesia. Furthermore, even within a single province, variation can be immense. It is somewhat disconcerting to note that overall poverty in *Kabupaten* Bangkalan $(M_{0}=0.07)$ can be seven times higher than in *Kota* Surabaya $(M_{0}=0.01)$, even though they belong to the same East Java province and are separated by no more than a 90-min drive.

The maps in Fig. 7 further reveal that the spatial patterning of poverty incidence tends not to match that of poverty intensity (Pearson’s $\rho =0.40$). In contrast to a cross-national pattern reported in the Human Development Report 2010 (UNDP 2010: 98), the incidence of poverty among districts in Indonesia does not seem to be linearly correlated with its intensity (the right panel of Fig. 8). Finally, also evident from this district-level analysis is the fact that urban areas tend to dominate rural areas. It is obvious that most of the top 10 least-deprived districts listed in Table 4 are municipalities (notice the *Kota* prefix) that, in general, have a higher level of urbanicity than ordinary districts (the *Kabupaten*). This is consistent with the result obtained earlier in Fig. 4.

### Inequality Among the Poor and Across Subgroups

**Table 5:** Measures of inequality

Inequality across…	*I*(2003)	*I*(2013)	$\Delta I^{*}$
Individual	0.098	0.076	0.022
Urban/rural	0.006	0.000	0.006
Gender	0.000	0.000	0.000
Island	0.003	0.000	0.003
Province	0.011	0.001	0.010
District	0.023	0.003	0.020

$^*$ All changes are statistically significant at 5 % level

Thus far, we have analysed the trend of multidimensional poverty in Indonesia by looking at the overall $(M_{0})$ and partial indices (*H*, *A*) as well as by decomposing the indices into relevant geographical or social subgroups. We found that there was an unambiguous poverty reduction between 2003 and 2013, both nationally and sub-nationally. But, with such an improvement, questions of distribution arise. Did the progress benefit the poorest of the poor? Has poverty reduction over the last decade been shared uniformly across population subgroups that make up Indonesian society?

In order to evaluate these, in Table 5 we calculate an inequality index (*I*) using the method proposed by Seth and Alkire (2014). This index is bounded between zero and one, capturing a state of complete equality up to that of complete inequality. It turns out that the reduction of multidimensional poverty in Indonesia within the last 11 years has been accompanied by an amelioration of the distribution of deprivations among the poor. The among-the-poor inequality decreased statistically significantly from 0.098 in 2003 to 0.076 in 2013, indicating inclusive progress. Similarly, the data show that there has been a convergence in poverty, meaning that poorer subgroups improved faster than the less poor. The disparity across subgroups has gone down for all relevant groupings (urban/rural, gender, island, province and district) over the 2003–2013 period. Finally, also evident from Table 5 is the fact that, in Indonesia, spatial inequality seems to matter more than gender or urban/rural inequality.

## Conclusion

Applying the Alkire–Foster method of multidimensional poverty measurement to the National Socio-economic Survey (Susenas) data of Indonesia, this study estimates the extent and investigates the regional as well as the temporal patterns of multidimensional poverty in Indonesia from 2003 to 2013. An Indonesian version of multidimensional poverty index (MPI) is developed through an augmentation of the existing consumption poverty measure with information on health and education that are represented by indicators of illness episode, morbidity, completion of primary school, and literacy.

It is found that, irrespective of the poverty cut-offs or weights specified, there was an unambiguous multidimensional poverty reduction over the last decade at both national and sub-national levels. About half (48 %) of Indonesian adults were multidimensionally poor in 2003 and, collectively, they experienced about one-fifth (0.19) of the total possible deprivations that the society could experience. In 2013, the situation was unmistakably better: only one in ten adults (11 %) was identified as multidimensionally poor, while the overall poverty figure fell to 0.04 (78 % reduction). The data suggest that the rate of poverty reduction was faster in the 2003–2008 period (60 %) than in 2008–2013 (44 %).

With the exceptions of rural areas and the Nusa Tenggara islands, there was minimal improvement with regard to the average deprivations experienced by the poor (intensity); overall poverty reduction was driven mainly by the decline in poverty incidence. It is further found that, when the overall measure is broken down into its dimensional constituents, income deprivation remains the main contributor to multidimensional poverty (60–70 %), albeit with a 2 % rate of decrease annually. Also estimated in the national-level analysis is the mismatch between income and multidimensional poverty identification. Results show that approximately 3 % of adult Indonesians (4.5 million individuals in 2013) would be classified as non-poor if poverty identification did not take into account deprivations in health and education. This figure could be as high as 7–17 % (11–26 million), depending on how much importance is assigned to schooling and/or illness episode indicators.

In an attempt to gain a more complete understanding of joint deprivation, the overall poverty measure is broken down by relevant population sub-groups. The data show that for each year from 2003 to 2013, multidimensional poverty was unambiguously higher in rural than in urban areas, but the gap between them has been progressively narrowing thanks to substantial improvement in both the incidence *and* the intensity of poverty in rural areas (rural-to-urban poverty ratio was 1.53 in 2003 vs. 1.25 in 2013). The data further reveal that Indonesian women are not unambiguously more deprived than men, although they appeared to have slightly more deprivations on average in the 2003–2007 period. Nevertheless, we cannot ascertain whether Indonesia has fared well in terms of gender equality because we cannot disentangle fully the information of women’s income using household expenditure data available at present.

In contrast to the clear trend seen in urban/rural and gender decompositions, we found only faint dominance in between-island comparisons over the 11-year period. It is only from 2010 onwards that it can be asserted with statistical confidence that poverty is unambiguously higher in Papua, Maluku and Nusa Tenggara (or lower in Kalimantan) than anywhere else in the archipelago. Even so, it is still important to note that such between-island comparisons mask a substantial amount of within-island and between-district variations, echoing both Ilmma and Wai-Poi (2014) and Sumarto et al. (2014). While five out of the ten poorest districts in 2013 are indeed located in Papua, three of them are in Sumatra and the other two are in Java, neither of which are thought of as places with extreme poverty. Analysis at the district level further reveals that, departing from the pattern observed in a cross-national study (UNDP 2010: 98), the intensity of poverty among districts in Indonesia does not seem to be related in a linear way to its incidence.

When the distribution of deprivations among the poor is studied, it is found that between 2003 and 2013, there were statistically significant improvements in terms of inequality among the poor and disparity across subgroups. The data show that poorer subgroups progress faster than the less poor, irrespective of the social or geographical groupings considered (converging subgroup poverty level). This finding indicates that the progress achieved within the last 11 years is relatively inclusive, although it should be noted that the between-district inequality within the Indonesian archipelago remains striking.

Overall, these trends are comparable to those obtained from recent consumption poverty evaluations conducted by Ilmma and Wai-Poi (2014) and Sumarto et al. (2014), highlighting the fact that, even a decade after a ‘big-bang’ decentralisation (Hill 2014) was initiated, spatial inequity remains a serious challenge for Indonesia. It has been argued that the immense variation in poverty levels across districts reflects heterogeneity in the ‘capacity and resources of local governments to develop and implement poverty reduction strategies, and to quickly provide good public services’ (Sumarto et al. 2014: 310). Only competent local government can formulate sound development plans, allocate budgets efficiently, and deliver public services effectively. Therefore, there is plenty of room for local administrators to learn lessons from the top-performing districts (Maharani and Tampubolon 2014).

While this study has presented a thorough investigation into the state of multidimensional poverty in Indonesia over the last decade, it is inevitably bound by several limitations. Firstly, the present study is unable to include children and adolescents younger than 18 years old in the analysis because information on the relevant dimensions of their well-being (Trani et al. 2013) are not available in Susenas survey. Secondly, the health indicators used in this study (illness episode and morbidity) are weak and by no means comparable to the indicators stipulated in the Millennium Development Goals (malnutrition). Thirdly, with the absence of preference data obtained from large-scale participatory study, the trade-offs between social indicators used in this study are entirely normative. In addition, although the measurement of chronic multidimensional poverty under the Alkire–Foster methodology has recently become feasible (Alkire et al. 2014), this study was unable to make use of it due to the cross-sectional nature of Susenas survey. It is indeed indisputable that future poverty evaluations would benefit from the availability of more comprehensive micro-data.

Even with these limitations, the study still contributes to the literature in at least three ways. First, using nationally representative survey data from Indonesia, the present study shows that the conventional measure of income poverty is not comprehensive. The Indonesian data reveal that income poverty only weakly correlates with deprivations in the domains of health and education, confirming the findings documented in other Asian (Ranis and Stewart 2012; Santos 2013; Yu 2013), African (Batana 2013; Klasen 2000), European (Brandolini and D’Alessio 1998; Whelan et al. 2004) and Latin American (Battison et al. 2013) countries. This may motivate future assessment of multidimensional poverty in other parts of the world.

Second, in using consumption expenditure data as the indicator of income, this study allows the poverty measure to become more sensitive to economic fluctuations than the current version of the international MPI (UNDP 2010), which uses asset ownership as a proxy for deprivation in living standards. This not only addresses one of the criticisms of the MPI (Ravallion 2010: 11), but also makes the MPI more comprehensible to Indonesians, who have for decades been accustomed to the conceptualisation of poverty as a consumption shortfall in essential goods and services.

Finally and most importantly, the present study demonstrates the feasibility of adapting the Alkire–Foster methodology to the Indonesian context using an existing official data source that has been in production since the 1960s (Surbakti 1995). Because the data are readily available, and the proposed multidimensional poverty measure presented here makes identification of multiply-deprived Indonesians possible, the MPI could nicely complement the existing indices that are routinely reported by the Indonesian Statistical Bureau (BPS). The new measure is suitable as a tool for monitoring the progress of national development, and could also be used as a device for prioritising investment projects or other forms of intervention that are funded by transfers from central to local governments (see Salazar et al. 2013 for a recent proposal in Colombia). With the demonstrated novelty, feasibility and utility of the Alkire–Foster method, policy makers should now more than ever want to incorporate the idea of poverty as an experience of multiple deprivations into the discourse of national development.

## References

[CR1] ADB. (2014). *Key indicators for Asia and the Pacific 2014*. Asian Development Bank, Manila, Philippines, Ch. Poverty in Asia: A Deeper Look.

[CR2] Alkire, S. (2011). *Multidimensional poverty and its discontents*. Oxford Poverty & Human Development Initiative (OPHI) Working Paper No. 46. http://www.ophi.org.uk/multidimensional-poverty-and-its-discontents/

[CR3] Alkire, S., Apablaza, M., Chakravarty, S. R., & Yalonetzky, G. (2014). *Measuring chronic multidimensional poverty: A counting approach*. Oxford Poverty & Human Development Initiative (OPHI) Working Paper No. 75. http://www.ophi.org.uk/measuring-chronic-multidimensional-poverty-a-counting-approach/

[CR4] Alkire S, Foster J (2011). Counting and multidimensional poverty measurement. Journal of Public Economics.

[CR5] Alkire S, Foster J (2011). Understandings and misunderstandings of multidimensional poverty measurement. Journal of Economic Inequality.

[CR6] Alkire S, Foster J, Santos ME (2011). Where did identification go?. Journal of Economic Inequality.

[CR7] Alkire, S., Roché, J. M., & Summer, A. (2013). *Where do the world’s multidimensionally poor people live*? Oxford Poverty & Human Development Initiative (OPHI) Working Paper No. 61. http://www.ophi.org.uk/where-do-the-worlds-multidimensionally-poor-people-live/

[CR8] Alkire S, Santos ME (2013). A multidimensional approach: Poverty measurement & beyond. Social Indicators Research.

[CR9] Alkire S, Santos ME (2014). Measuring acute poverty in the developing world: Robustness and scope of the multidimensional poverty index. World Development.

[CR10] Alkire, S., & Vaz, A. (2014). *Multidimensional poverty dynamics with time series and panel data*. OPHI Summer School on Multidimensional Poverty Analysis. http://www.ophi.org.uk/wp-content/uploads/3-SS14-Analysis_Over_Time.pdf

[CR11] Atkinson AB (2003). Multidimensional deprivation: Contrasting social welfare and counting approaches. Journal of Economic Inequality.

[CR12] Batana YM (2013). Multidimensional measurement of poverty among women in Sub-Saharan Africa. Social Indicators Research.

[CR13] Battison D, Cruces G, Lopez-Calva LF, Lugo MA, Santos ME (2013). Income and beyond: Multidimensional poverty in six Latin American countries. Social Indicators Research.

[CR14] Bourguignon, F., Bénassy-Quéré, A., Dercon, S., Estache, A., Gunning, J. W., Kanbur, R., et al. (2008). *Millennium development goals at midpoint: Where do we stand and where do we need to go*? European Commission Report. http://ec.europa.eu/development/icenter/repository/mdg_paper_final_20080916_en.pdf

[CR15] Bourguignon F, Chakravarty SR (2003). The measurement of multidimensional poverty. Journal of Economic Inequality.

[CR16] BPS. (2015a). *Jumlah dan Persentase Penduduk Miskin, Garis Kemiskinan, Indeks Kedalaman Kemiskinan (P1), dan Indeks Keparahan Kemiskinan (P2) Menurut Provinsi, 2007–2009 (Maret), 2010–2011, 2012 (Maret dan September)*. http://www.bps.go.id/webbeta/frontend/linkTabelStatis/view/id/1489

[CR17] BPS. (2015b). *Konsep Indeks Pembangunan Manusia* (online). Accessed February 27, 2015. http://www.bps.go.id/webbeta/frontend/Subjek/view/id/26#subjekViewTab1

[CR18] BPS. (2015c). *Konsep Kemiskinan* (online). Accessed February 27, 2015. http://www.bps.go.id/webbeta/frontend/Subjek/view/id/23#subjekViewTab1

[CR19] BPS, Bappenas, UNDP. (2004). *Ekonomi dari Demokrasi: Membiayai Pembangunan Manusia Indonesia*. Laporan Pembangunan Manusia Indonesia 2004.

[CR20] Brandolini, A., & D’Alessio, G. (1998). *Measuring well-being in the functioning space*. Research note. http://www.iberoamericana.edu.mx/humanismocristiano/seminario_capability/pdf/3.pdf

[CR21] Decancq K, Lugo MA (2013). Weights in multidimensional indices of wellbeing: An overview. Econometric Reviews.

[CR22] Filmer D, Pritchett LH (2001). Estimating wealth effects without expenditure data—or tears: An application to educational enrollments in states of India. Demography.

[CR23] Foster, J. (2007). *A report on Mexican multidimensional poverty measurement*. Oxford Poverty & Human Development Initiative (OPHI) Working Paper No. 40. http://www.ophi.org.uk/a-report-on-mexican-multidimensional-poverty-measurement/

[CR24] Foster J, Greer J, Thorbecke E (1984). A class of decomposable poverty measures. Econometrica.

[CR25] Grossman M (1972). On the concept of health capital and the demand for health. Journal of Political Economy.

[CR26] Hill H (2014). Regional dynamics in a decentralized Indonesia.

[CR27] ICF International. (2012). *Demographic and health surveys* (online). Accessed February 27, 2015. http://dhsprogram.com/data/

[CR28] Ilmma A, Wai-Poi M, Hill H (2014). Patterns of regional poverty in the new Indonesia. Regional dynamics in a decentralized Indonesia.

[CR29] Klasen S (2000). Measuring poverty and deprivation in South Africa. Review of Income and Wealth.

[CR30] Maharani A, Tampubolon G (2014). Has decentralisation affected child immunisation status in Indonesia?. Global Health Action.

[CR31] MPR RI. (2011). *Undang–Undang Dasar Negara Republik Indonesia Tahun 1945*. Majelis Permusyawaratan Rakyat Republik Indonesia. http://www.mpr.go.id/pages/produk-mpr/uud-nri-tahun-1945

[CR32] Nazara, S. (2010). *The informal economy in Indonesia: Size, composition and evolution*. The International Labour Organization Working Paper. http://www.ilo.org/jakarta/whatwedo/publications/WCMS_145781/lang--en/index.htm

[CR33] RAND. (2007). *IFLS-4 survey description* (online). Accessed May 1, 2014. http://www.rand.org/labor/FLS/IFLS/ifls4.html

[CR34] RAND. (2014). *Indonesia Family Life Survey East (IFLS EAST)* (online). Accessed February 27, 2015. http://www.rand.org/labor/FLS/IFLS/ifls-east.html

[CR35] Ranis G, Stewart F (2012). Success and failure in human development, 1970–2007. Journal of Human Development and Capabilities.

[CR36] Ravallion, M. (2010). *Mashup indices of development*. World Bank Policy Research Working Paper No. 5432. http://elibrary.worldbank.org/doi/abs/10.1596/1813-9450-5432

[CR37] Ravallion M (2011). On multidimensional indices of poverty. Journal of Economic Inequality.

[CR38] Ravallion M, Chen S, Sangraula P (2009). Dollar a day revisited. World Bank Economic Review.

[CR39] Roche JM (2013). Monitoring progress in child poverty reduction: Methodological insights and illustration to the case study of Bangladesh. Social Indicators Research.

[CR40] Sahn DE, Stifel D (2003). Exploring alternative measures of welfare in the absence of expenditure data. Review of Income and Wealth.

[CR41] Salazar, R. C. A., Diaz, B. Y., & Pinzon, R. P. (2013). *A counting multidimensional poverty index in public policy context: The case of Colombia*. Oxford Poverty & Human Development Initiative (OPHI) Working Paper No. 62. http://www.ophi.org.uk/a-counting-multidimensional-poverty-index-in-public-policy-context-the-case-of-colombia/

[CR42] Santos ME (2013). Tracking poverty reduction in Bhutan: Income deprivation alongside deprivation in other sources of happiness. Social Indicators Research.

[CR43] Santos, M. E., & Ura, K. (2008). *Multidimensional poverty in Bhutan: Estimates and policy implications*. Oxford Poverty & Human Development Initiative (OPHI) Working Paper No. 14. http://www.ophi.org.uk/working-paper-number-14/

[CR44] Schultz PT, Tansel A (1997). Wage and labor supply effects of illness in Côte d’Ivoire and Ghana: Instrumental variable estimates for days disabled. Journal of Development Economics.

[CR45] Sen A (1976). Poverty: An ordinal approach to measurement. Econometrica.

[CR46] Sen A (1985). Commodities and capabilities.

[CR47] Seth, S., & Alkire, S. (2014). *Did poverty reduction reach the poorest of the poor? Assessment methods in the counting approach*. Oxford Poverty & Human Development Initiative (OPHI) Working Paper No. 77. http://www.ophi.org.uk/did-poverty-reduction-reach-the-poorest-of-the-poor-assessment-methods-in-the-counting-approach/

[CR48] Strauss J, Beegle K, Dwiyanto A, Herawati Y, Pattinasarany D, Satriawan E, Sikoki B, Sukamdi, Witoelar F (2004). Indonesian living standards before and after the financial crisis: Evidence from the Indonesia Family Life Survey.

[CR49] Sumarto S, Vothknecht M, Wijaya L, Hill H (2014). Explaining regional heterogeneity of poverty: Evidence from a decentralized Indonesia. Regional dynamics in a decentralized Indonesia.

[CR50] Surbakti P (1995). Indonesia’s National Socio-economic Survey: A continual data source for analysis on welfare development.

[CR51] Székely, M. (2003). *Lo que dicen los pobres*. Cuadernos de desarrollo humano No. 13. Secretaría de Desarrollo Social, México.

[CR52] Thomas D, Witoelar F, Frankenberg E, Sikoki B, Strauss J, Sumantri C, Suriastini W (2012). Cutting the costs of attrition: Results from the Indonesia Family Life Survey. Journal of Development Economics.

[CR53] Townsend, P. (1979). *Poverty in the United Kingdom*. London: Allen Lane and Penguin Books. http://www.poverty.ac.uk/free-resources-books/poverty-united-kingdom

[CR54] Trani J-F, Biggeri M, Mauro V (2013). The multidimensionality of child poverty: Evidence from Afghanistan. Social Indicators Research.

[CR55] UNDP (2010). The real wealth of nations: Pathways to human development.

[CR56] UNDP. (2014). *Why is the MPI better than the Human Poverty Index (HPI) which was previously used in the Human Development Reports?* Frequently Asked Questions, United Nations Development Programme (online). Accessed February 27, 2015. http://hdr.undp.org/en/faq-page/multidimensional-poverty-index-mpi#t295n138

[CR57] UNSD. (2014). *Purchasing power parities (PPP) conversion factor, local currency unit to international dollar*. United Nations Statistics Division. http://mdgs.un.org/unsd/mdg/SeriesDetail.aspx?srid=699

[CR58] Ura, K., Alkire, S., Zangmo, T., & Wangdi, K. (2012). *An extensive analysis of GNH index*. Centre for Bhutan Studies. http://www.grossnationalhappiness.com/wp-content/uploads/2012/10/An%20Extensive%20Analysis%20of%20GNH%20Index.pdf

[CR59] van de Walle, D. (1988). On the use of the susenas for modelling consumer behaviour. *Bulletin of Indonesian Economic Studies*, *24*(2), 107–121.

[CR60] Whelan CT, Layte R, Maître B (2004). Understanding the mismatch between income poverty and deprivation: A dynamic comparative analysis. European Sociological Review.

[CR61] Yu J (2013). Multidimensional poverty in China: Findings based on the CHNS. Social Indicators Research.

